# Long-Term Suppressive Antimicrobial Therapy in Prosthetic Vascular Graft Infection: A Retrospective Evaluation of a Cohort of Patients Enrolled at Tor Vergata Hospital in Rome

**DOI:** 10.1093/ofid/ofag327

**Published:** 2026-06-05

**Authors:** Alessandra Imeneo, Drieda Zaçe, Fabio Massimo Oddi, Grazia Alessio, Ludovica Ferrari, Tiziana Mulas, Angela Crea, Vincenzo Malagnino, Andrea Ascoli Marchetti, Arnaldo Ippoliti, Marco Iannetta, Loredana Sarmati

**Affiliations:** Infectious Diseases, Department of Systems Medicine, Tor Vergata University, Rome, Italy; Clinical and Research Infectious Diseases Department, National Institute for Infectious Diseases “L. Spallanzani” IRCCS, Rome, Italy; Infectious Diseases, Department of Systems Medicine, Tor Vergata University, Rome, Italy; Vascular and Endovascular Surgery, Department of Biomedicine and Prevention, Tor Vergata University, Rome, Italy; Infectious Diseases, Department of Systems Medicine, Tor Vergata University, Rome, Italy; Clinical and Research Infectious Diseases Department, National Institute for Infectious Diseases “L. Spallanzani” IRCCS, Rome, Italy; Infectious Diseases, Department of Systems Medicine, Tor Vergata University, Rome, Italy; Infectious Disease Clinic, Policlinico Tor Vergata, Rome, Italy; Infectious Disease Clinic, Policlinico Tor Vergata, Rome, Italy; Infectious Diseases, Department of Systems Medicine, Tor Vergata University, Rome, Italy; Infectious Disease Clinic, Policlinico Tor Vergata, Rome, Italy; Vascular and Endovascular Surgery, Department of Biomedicine and Prevention, Tor Vergata University, Rome, Italy; Vascular and Endovascular Surgery, Department of Biomedicine and Prevention, Tor Vergata University, Rome, Italy; Infectious Diseases, Department of Systems Medicine, Tor Vergata University, Rome, Italy; Infectious Disease Clinic, Policlinico Tor Vergata, Rome, Italy; Infectious Diseases, Department of Systems Medicine, Tor Vergata University, Rome, Italy; Infectious Disease Clinic, Policlinico Tor Vergata, Rome, Italy

**Keywords:** graft, long-acting antibiotics, PET/CT scan, prosthetic vascular graft infection (PVGI), suppressive antimicrobial therapy (SAT)

## Abstract

**Background:**

Prosthetic vascular graft infection (PVGI) is a rare but life-threatening complication. Graft replacement is the only curative option, but often unfeasible due to high mortality risk. Suppressive antimicrobial therapy (SAT) offers a viable alternative, though evidence on regimens and duration is limited.

**Methods:**

We conducted a retrospective study, reviewing records of PVGI patients during 2014–2025 aiming to evaluate the efficacy of SAT. SAT was considered effective when allowing infection control under treatment; patients were defined as cured if no relapse occurred after at least 6 months from discontinuation. Patients were classified according to the Management of Aortic Graft Infection Collaboration criteria and managed by a multidisciplinary team.

**Results:**

Thirty patients met the inclusion criteria; 5 underwent graft removal and 25 underwent SAT. Infections were diagnosed a median of 90 [30–1095] days postsurgery. Fever occur in 15/25 (60%) and abscess in 12/25 (48%) patients. Microbiological diagnosis was achieved in 17/25 (68%) cases. A long-acting antibiotic (LAA) was used in 6/25 (24%), with successful discontinuation in 3/6. Drug adverse events occurred in 8/25 (32%) patients and treatment failure was observed in 11 among all antibiotic regimens analyzed. Infection control during SAT was achieved in 10 (40%) patients. Ten patients discontinued SAT and 6/10 (60%) remained relapse-free after more than 6 months of follow-up.

**Conclusions:**

SAT is a valuable option for high-risk PVGI patients. LAA may represent an option. PET/CT remains key for diagnosis and monitoring. Further studies are needed to define optimal SAT duration and to improve long-term outcomes.

## Teaser

In prosthetic vascular infection (PVGI), prosthesis removal is the only curative option, but it is often associated with a high surgical risk. Suppressive antimicrobial therapy (SAT) represents a valid alternative. The safety and efficacy of SAT at a tertiary care center are here reported.

Prosthetic vascular graft infection (PVGI) is a rare but life-threatening complication of surgery, with an incidence of 0.4%–4% for traditional surgery and 0.1%–0.7% for endovascular repair [1–5].

According to the time of onset, PVGIs are classified as “early” if they occur within 4 months or “late” otherwise [3]. Early PVGIs result from intraoperative or postoperative contamination and are caused by virulent microorganisms such as *Staphylococcus aureus* or Gram-negative bacteria. Late PVGIs arise from aorto-enteric fistula, local infections or bacteremia and are typically caused by coagulase-negative staphylococci from skin flora or by polymicrobial infections, including some anaerobes and fungi [3, 6]. Early infections generally present as systemic disease with fever and leukocytosis, whereas late infections often have a more subclinical presentation, frequently associated with signs of complications, such as false aneurysm, gastrointestinal bleeding, hydronephrosis, or osteomyelitis [4, 7].

PVGI is an increasingly relevant clinical challenge, and the optimal management strategies are still debated. The European Society for Vascular Surgery recommends complete removal and replacement of the graft combined with antibiotic therapy, as the only curative approach [8]. However, this option is often unfeasible due to high surgical risk [3, 9]. Consequently, suppressive antimicrobial therapy (SAT), lasting at least 6–12 months or potentially lifelong [10–12], represents the most viable option for the majority of patients, allowing infection control and reducing the risk of relapse.

Randomized trials evaluating antibiotic strategies for PVGI are lacking. The existing literature reports considerable heterogeneity in treatment regimens and duration. Moreover, there has been a growing interest in the use of long-acting antibiotics (LAAs) in endovascular infections [13–16], although the optimal timing of dose administration remains under investigation.

Finally, a multidisciplinary approach, involving surgeons and infectious disease specialists, is strongly recommended to tailor the best management for each individual patient with PVGI.

The principal aim of the study was to evaluate the efficacy of SAT defined as infection control during treatment, with resolution of clinical signs of infection and radiological improvement or stability.

## METHODS

This study is reported according to strengthening the reporting of observational studies in epidemiology [17].

This retrospective observational study was conducted at the University Hospital Policlinico Tor Vergata in Rome, a tertiary care center with both vascular surgery and infectious diseases departments. Clinical records of patients evaluated at Infectious Diseases Clinic and/or Vascular Surgery Clinic from January 2014 to February 2025 were reviewed.

Inclusion criteria were adult patients with a graft (both traditional and endovascular) and concomitant PVGI according to Management of Aortic Graft Infection Collaboration (MAGIC) [3] criteria. Exclusion criteria were radiological evidence of concomitant heart valve infection (prosthetic or native) and complete surgical replacement of the graft.

Outcomes were retrospectively assessed based on the most recent available outpatient evaluation at the data of February 2025. For patients who had discontinued outpatient follow-up, telephone interviews were conducted, when possible, to obtain up-to-date clinical information. (See also Supplementary Figure 1).

After initial inpatient intravenous treatment, all antibiotic regimens prescribed after discharge, whether oral or intravenous, were considered as SAT. The primary outcome was the efficacy of SAT, defined as infection control during treatment, with resolution of clinical signs of infection and radiological improvement or stability. For this purpose, patients were categorized into 2 groups: those who achieved resolution of clinical signs and symptoms and radiological resolution or stability were classified as *improved*. In contrast, patients who experienced clinical and/or radiological progression of infection while on SAT were classified as *treatment failures*, requiring a modification or escalation of therapy.

The secondary outcomes included: (1) SAT tolerability, defined as the absence of drug adverse events necessitating a change in therapy; and (2) PVGI cure, defined as the absence of relapse during at least 6 months of follow-up after SAT discontinuation. Patients who discontinued SAT without experiencing a relapse were classified as *cured*, whereas those who experienced clinical and/or radiographic evidence of worsening infection were considered *relapsed*. Specifically, relapses were defined as the recurrence of symptoms present at onset and/or radiological signs suspicious for infection, with or without microbiological findings. However, in the absence of microbiological data, relapse and reinfection by a different microorganism could not be reliably differentiated. All deaths were recorded, regardless of their association with PVGI.

According to the MAGIC [3] criteria, PVGIs were stratified as either “suspected” or “diagnosed” and as “early” or “late.” PVGI was defined as “early” if it occurred within 4 months from the initial endovascular repair and “late,” otherwise. A PVGI was defined as “suspected” if a single major criterion or at least 2 criteria from different categories (clinical/surgical, radiological, or laboratory) were present. A PVGI was defined as “diagnosed” if one major criterion plus any criterion (major or minor) from another category was present.

For diagnosis, patients underwent clinical, microbiological and radiological evaluation. All patients had blood tests and blood cultures performed. Elevated inflammatory markers were defined as: white blood cells count >12.000/µL and/or C-reactive protein >5.00 mg/L and/or procalcitonin >0.5 ng/mL. Serial blood cultures, swab cultures or drainage fluid cultures were performed and interpreted as suggestive of infection rather than contamination, according to microbial findings and clinical signs. Microbiological isolates were considered contaminants if coagulase-negative staphylococci were isolated in a single broth only, or if skin flora bacteria were isolated from a swab or drainage fluid in the absence of signs or symptoms suggestive of infection.

According to clinical indication, a computed tomography (CT) scan and/or 18F-fluorodeoxyglucose (FDG) positron emission tomography (PET/CT) scan were performed. Major CT scan criteria suggestive of PVGI included the presence of an abscess, endoleak, fistula or reactive lymph nodes. Increased metabolic activity of the graft on PET/CT scan was considered suspicious for PVGI.

After initial inpatient intravenous treatment, a multidisciplinary approach, involving infectious disease and vascular/cardiac surgeons, was adopted to determine the indication for SAT in patients not eligible for surgical intervention. Patients who underwent successful surgical *source-control* and discontinued SAT following graft removal were excluded from SAT outcome analysis.

**Figure 1.**
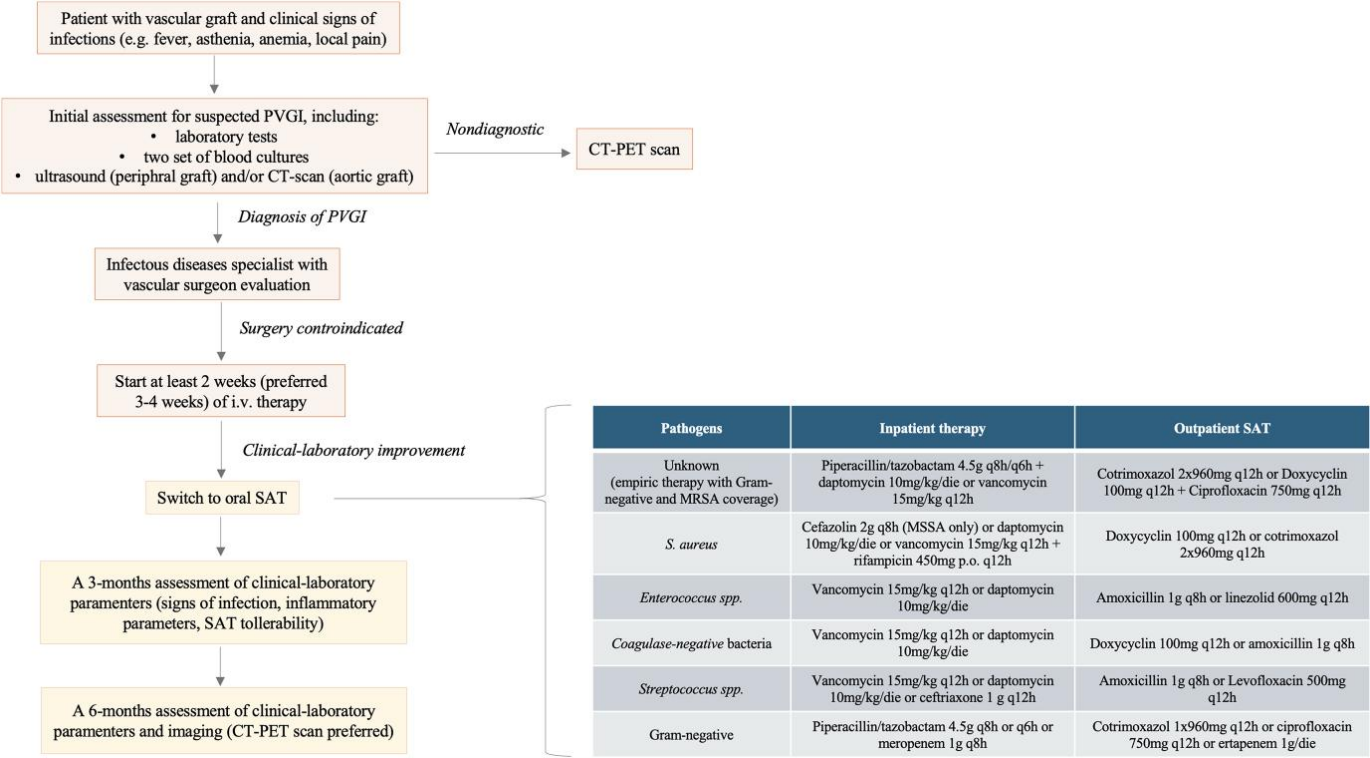
Diagnostic evaluation and assessment of PVGI. CT, computed tomography; PET, positron emission tomography; PVGI, prosthetic vascular graft infection; i.v., intravenous; SAT, suppressive antimicrobial treatment; MRSA, *Staphylococcus aureus* meticillino-resistant; q, interval between doses administration.

During SAT, patients were reassessed at 3 months after initiation and then every 6 months at our Infectious Diseases Clinic by a dedicated team, until treatment discontinuation. An algorithm for PVGI assessment is reported in Figure 1. Clinical signs and symptoms of infection, as well as inflammatory markers, were evaluated at each visit. Blood cultures were not routinely performed. Radiological assessments were conducted based on clinical indication, resulting in heterogeneous follow-up time.

For the analysis of adverse effects and treatment failures, all SAT regimens adopted in the cohort were considered. Side effects were monitored separately for each antibiotic regimen, acknowledging that some patients in the cohort underwent more than 1 treatment regimen. In combination therapy, antibiotics in each regimen were considered independently responsible for treatment failure; however, in the case of drug adverse effects, only the likely causative antibiotic (for which the adverse effect was reported as frequent) was considered responsible and therefore discontinued.

### Ethics

The study was approved by the Ethics Committee of Fondazione PTV Policlinico Tor Vergata (protocol number 346.24). All procedures were carried out in accordance with the Declaration of Helsinki (WMA 2013) and relevant guidelines and regulations. Given the retrospective and observational nature of the study, the requirement for patient informed consent was waived by the Ethics Committee.

### Statistical Analysis

Quantitative variables are presented as median and interquartile range (IQR), while categorical variables are presented as absolute frequency and percentages (%). Differences between groups were assessed with the Fisher's test for categorical data and the Wilcoxon rank sum test or Kruskal–Wallis test for continuous data as appropriate. The sensitivity of CT angiography, PET/CT and blood cultures was calculated separately for diagnosed and suspected PVGI, using the MAGIC criteria as the reference standard. For all the tests, the level of statistical significance was <0.05. Statistical analysis was performed with STATA v.15.0 software and JASP v.0.95.1.

## RESULTS

### Patients' Characteristics

The selection process of the 35 patients with PVGI enrolled during the study period is shown in Supplementary Figure 2. Five patients did not meet the MAGIC criteria and were excluded. Five out of 30 patients underwent complete removal of the endograft and were excluded from further analysis; therefore, a final population of 25 patients was included in the study. Among the 25 patients enrolled, one presented with aorto-enteric fistula; surgery was initially contraindicated due to high operative risk and SAT was started, after which he was lost to follow-up.

Demographic and clinical characteristics of the 25 enrolled patients are reported in Table 1. Among the overall population, a prevalence of male sex was observed (21/25, 84%), with a median age of 72 years [IQR 62–78]. The median Charlson comorbidity index was 3 [IQR 2–6], with cardiovascular disease (19/25, 76%), chronic kidney disease (6/25, 24%), diabetes mellitus (6/25, 24%) and pulmonary disease (5/25, 20%) being the most common comorbidities (see also Supplementary Table 1).

**Table 1.** Demographic and Clinical Characteristics of the Enrolled Population (25 Patients)

n°	Sex, Age	CCI	Type of Surgery	Site	Diagnosis	ΔT (Days) Surgery-PVGI	MAGIC Criteria	Early Complications	Symptoms	[18F] FDG PET/CT SUV Max
1	M 74	3	EVAR	Abdominal aorta	2014	90	D	Abscess	Anemia	Positive
2	M 82	4	EVAR	Aorto-iliac	2015	3650	D	Abscess, osteomyelitis	Pain	N.A.
3	M 70	3	EVAR	Abdominal aorta	2017	30	S	None	Fever	SUV 7.0
4	M 58	8	EVAR	Abdominal aorta	2018	N.A.	D	None	Pain	N.A.
5	M 51	1	open surgery	Ascending aorta	2019	30	D	Abscess, mediastinitis	Fever	SUV 6.1
6	M 78	6	FEVAR	Abdominal aorta	2019	270	D	Abscess	Fever	SUV 13.1
7	M 62	2	open surgery	Abdominal aorta	2019	1095	S	None	Fever, pain	SUV 10.7
8	M 76	7	EVAR	Abdominal aorta	2019	4745	S	None	Fever	SUV 5.5
9	M 83	5	BEVAR	Aorto-iliac	2020	120	D	Abscess, duodenal fistula	Pain	N.A.
10	M 64	2	open surgery	Ascending aorta and aortic arch	2020	6205	S	None	Fever	SUV 10.7
11	M 62	2	TEVAR	Thoracic aorta	2021	14	D	Abscess	Fever	N.A.
12	F 65	3	open surgery	Abdominal aorta	2021	7	D	Abscess	Wound dehiscence	SUV 2.8
13	M 78	3	open surgery	Ascending aorta and aortic arch	2022	2190	S	None	Fever	SUV 7.4
14	F 59	1	EVAR + stent	Aorto-iliac	2022	180	S	None	Fever, pain	SUV 3.35
15	F 60	4	TEVAR	Thoracic aorta	2022	30	S	None	Pain	SUV 5.8
16	M 78	9	TEVAR + EVAR	Left iliac artery	2022	90	D	Abscess	Anemia	SUV 4.5
17	F 78	5	TEVAR + EVAR + Bentall	Thoraco-abdominal aorta	2022	30	D	Abscess, spondylodiscitis	Pain	SUV 7.6
18	M 54	3	TEVAR	Thoracic aorta	2023	2920	D	Abscess	Fever	SUV 9.7
19	M 73	6	Ch-EVAR	Aorto-iliac	2023	1095	S	None	Pain	SUV 12.0
20	M 84	9	EVAR	Aorto-iliac	2023	30	D	Abscess, spondylodiscitis	Pain	N.A.
21	M 76	10	Stent	Right iliac artery	2023	14	S	None	Fever, pain	SUV 9.9
22	M 72	5	TEVAR + EVAR	Thoraco-abdominal aorta	2023	90	D	Abscess	Fever, pain	SUV 9.0
23	M 62	2	open surgery	Ascending aorta and aortic arch	2023	30	S	None	Fever	N.A.
24	M 76	3	TEVAR + EVAR	Thoraco-abdominal aorta	2024	330	S	None	Fever	SUV 9.0
25	M 66	3	EVAR	Abdominal aorta	2024	N.A.	D	None	Fever, pain	N.A.

PVGI were classified as “suspected” or “diagnosed” according to *Lyons OT et al Diagnosis of Aortic Graft Infection: A Case Definition by the Management of Aortic Graft Infection Collaboration (MAGIC). Eur J Vasc Endovasc Surg. 2016.*

B, blood sample; BEVAR, branched endovascular aneurysm repair; CCI, Charlson Comorbidity Index; Ch-EVAR, chimney endovascular aneurysm repair; D, diagnosed infection; DR, drainage sample; EVAR, endovascular aneurysm repair; F, female; FDG, fluorodeoxyglucose; FEVAR, fenestrated endovascular aneurysm repair; I, intraoperative specimen; M, male; NA, not accomplished; PET, positron emission tomography; PVGI, Prosthetic vascular graft infection; S, suspected infection; SUV, maximal standardized uptake value; TEVAR, thoracic endovascular aneurysm repair; W, wound swab; ΔT Surgery-PVGI, time between vascular graft implantation and diagnosis of infection.

### PVGI Characteristics

Initial endovascular repair was most frequently performed for aneurysms (15/25, 60%) or aortic dissections (5/25, 20%). The surgical procedures and type of endograft are reported in Table 1. Of the procedures, 13/25 (52%) were elective and 12/25 (48%) were emergency procedures, mostly due to aortic dissection or aneurysm rupture. In our cohort, 14/25 (56%) were early infections and 11/25 (44%) were late infections. Endovascular surgery (19/25, 76%) was more common than traditional surgery (6/25, 24%), as shown in Table 1.

All included patients had vascular graft infection of the aorta or iliac artery: 15/25 (60%) had abdominal involvement, 7/25 (28%) patients had thoracic involvement and 3/25 (12%) had both. Patients over 65 years showed a higher incidence of abdominal PVGI (80% vs 20%, *P* value .01). Fourteen out of the twenty-five (56%) patients met the MAGIC criteria for definite PVGI and 11/25 (44%) for suspected PVGI. Two patients were diagnosed with PVGI due to graft insertion in an infected site (previous aortitis).

PVGI was diagnosed after a median of 90 days [IQR 30–1095] from the initial surgery. Symptoms at presentation included fever (15/25, 60%), local pain (12/25, 48%), anemia (2/25, 8%). Most patients (21/25, 84%) had elevated inflammatory markers on initial blood tests. Twelve (48%) patients experienced complications such as abscess (12/25, 48%), bone infection (3/25, 12%), aorto-enteric fistulae (1/25, 4%) or mediastinitis (1/25, 4%).

### Diagnosis of PVGI

CT and PET/CT scans were the preferred imaging at diagnosis (Table 1). Among patients with diagnosed PVGI, 9/12 had CT findings suggestive of infection (sensitivity of 75%). In patients with suspected PVGI, 4/8 had suggestive CT findings (sensitivity of 50%). PET/CT showed a 100% sensitivity in both diagnosed PVGI (8/8) and suspected PVGI (10/10). Three (12%) patients (1 suspected PVGI and 2 diagnosed PVGI) underwent leukocyte scintigraphy, all of which were negative.

**Figure 2.**
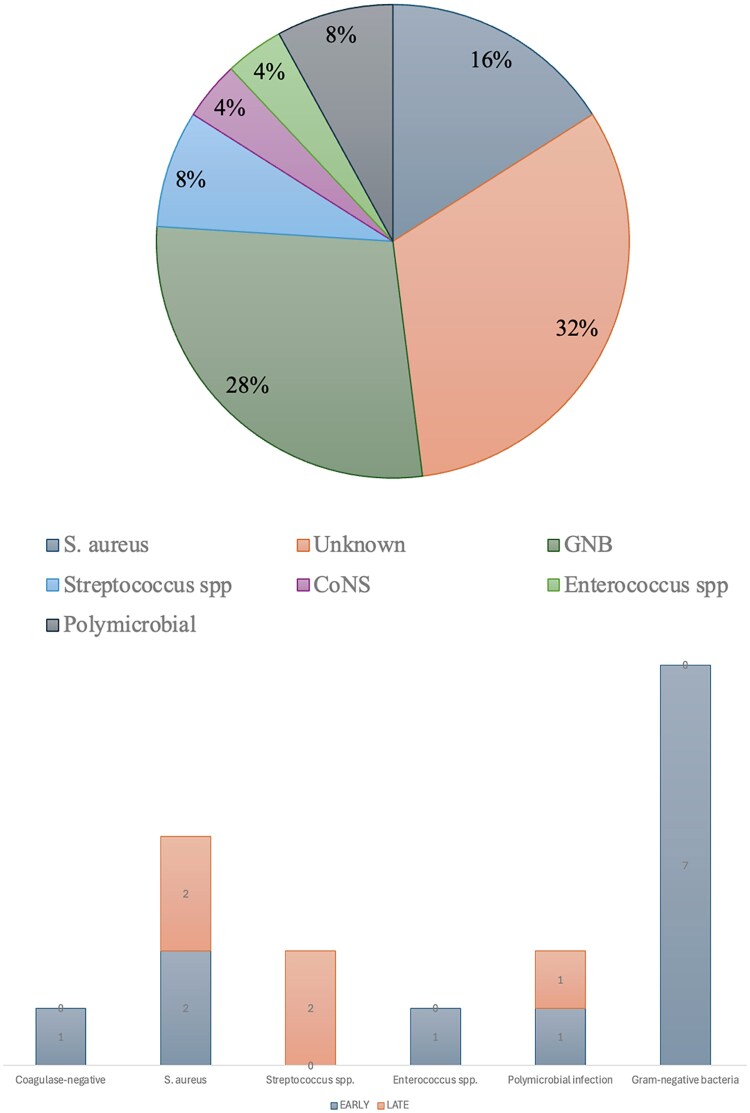
Distribution of the isolates overall and by subgroups (*early* vs *late* PVGI). GNB, Gram-negative bacteria; GPB, Gram-positive bacteria; CoNS, coagulase-negative Staphylococci. Among the 25 patients enrolled, the causative pathogen was identified in 17 patients. Monomicrobial GPB infections were caused by: *S. aureus* (4), of which one methicillin-resistant; *S. capitis* (1), *S. sanguinis* (1), *S. gallolyticus* (1), *E. faecalis* (1). Monomicrobial GNB infections were caused by: *E. coli* (2), *P. aeruginosa* (2), *E. cloacae* (1), *S. enteritidis* (1), *S. marcescens* (1). Polymicrobial infections were caused by: *S. mitis* and *S. constellatus* (1); *S. hominis* and *M. luteus* (1).

At least 1 causative organism was identified in 17/25 (68%) patients (Figure 2), primarily through blood cultures (15/17, 88%). The remaining 2 patients had organism identification performed using intraoperative samples and wound swabs. In patients with diagnosed PVGI, blood culture sensitivity was 69% (9/13), while in suspected PVGIs it was 54% (7/13). Microbiological diagnosis was more frequently achieved in early infections (86% vs 45%, *P* value .03).

### Microbiological Isolates

PVGI was caused by monomicrobial Gram-positive bacteria (GPB) in 8/17 (47%) cases and by monomicrobial Gram-negative bacteria (GNB) in 7/17 (41%) cases; 2/17 (12%) infections were polymicrobial. Excluding polymicrobial infections*, S. aureus* was the most common pathogen (4/15, 27%), including 1 *Methicillin-Resistant Staphylococcus Aureus* (MRSA). In 8 patients, the causative organism was not identified.

### Treatment

All patients received an initial empiric intravenous antibiotic, typically a beta-lactam combined with an anti-MRSA agent (vancomycin or daptomycin); then, once the causative organism was identified, antibiotic treatment was targeted based on the results of the antimicrobial susceptibility tests. Intravenous antimicrobial therapy had a median duration of 23 days [IQR 16–38].

The characteristics of the 25 patients who underwent SAT are reported in Table 2.

**Table 2.** Treatment of the Prosthetic Vascular Graft Infection (PVGI) and Outcome of the Population (25 Patients)

n°	Isolates	Overall Treatment (Days)	Inpatient I.v. Therapy (Days)	1° SAT	Duration (Days) and Reason For Switch	2° SAT	Duration (Days) and Reason for Switch	3° SAT	Duration (Days) and Reason For Switch	4° SAT	Outcome (FU After SAT Discontinuation)
1	*S. aureus*	1841	9	TEI	1832 **Stop**	…	…	…	…	…	Recurrence Death
2	*…*	N.A.	N.A.	AMC + LVX	1096 CF, RF	STX + CIP	246 Renal	AMC + CIP	…	…	Death
3	*S. marcescens*	362	28	CIP	334 **Stop**	…	…	…	…	…	Suspension (6 years)
4	*S. enteritidis*	21	21	AMC	N.A. **Stop**	…	…	…	…	…	Recurrence Death
5	*P. aeruginosa*	1399	89	CIP	1310 **Stop**	…	…	…	…	…	Suspension (20 months)
6	*S. aureus*	125	65	CLI	8 Rash	DOX + RIF	65 Nausea	DOX	117 **Stop**	…	Suspension (5 year)
7	*…*	281	23	AMC	21 CF, LF	CIP + DAL (16)	237 **Stop**	…	…	…	Suspension (4 years)
8	*S. gallolyticus*	32	32	CRO	…	…	…	…	…	…	Death
9	*S. mitis,* *S.constellatus*	14	14	LVX	…	…	…	…	…	…	Lost at FU
10	*S. aureus*	N.A.	N.A.	TEI	…	…	…	…	…	…	Death
11	*S. aureus*	Ongoing	74	STX	238 Renal	MIN	511 Ph	DOX	…	…	Ongoing treatment
12	*P. aeruginosa*	336 Ongoing	16	CIP	320 **Stop**	CIP	…	…	…	…	Recurrence Ongoing treatment
13	*S. sanguinis*	Ongoing	42	AMC	92 LF	AMC + DAL (4)	86 S	AMC	…	…	Ongoing treatment
14	*…*	Ongoing	19	AMC	47 RF	DOX	100 RF	DOX + AMC	79 Ph LF	DAL (N.A.)	Ongoing treatment
15	*S. hominis, M. luteus*	Ongoing	14	AMC	67 RF	TEI	…	…	…	…	Ongoing treatment
16	*…*	97	20	DAP	13 S	AMC	64 RF	DOX	…	…	Death
17	*E. faecalis*	N.A. Ongoing	N.A.	AMC + DAL (7)	163 **Stop**	AMC + ORT (1)	19 Tremor	AMC + LZD	31 **Stop**	AMC	Recurrence Ongoing treatment
18	*…*	291	34	STX	33 LF	AMC + DAL	224 **Stop**	…	…	…	Suspension (1 year)
19	*…*	Ongoing	8	DOX	…	…	…	…	…	…	Ongoing treatment
20	*E. coli*	Ongoing	52	STX	43 RF	ERT	Neutropenia	…	…	…	Ongoing treatment
21	*E. cloacae*	Ongoing	21	STX	29 renal	CIP	…	…	…	…	Ongoing treatment
22	*E.coli*	Ongoing	27	AMC	11 CF, RF	ERT	…	…	…	…	Ongoing treatment
23	*S. capitis*	125	125	STX	…	…	…	…	…	…	Lost at FU
24	*…*	28	13	ERT	15 S	STX	…	…	…	…	Death
25	…	167	34	CRO + DAL (4)	133 Hepatic **Stop**	…	…	…	…	…	Suspension (8 months)

The overall number of LAA doses administered is reported in branches. All known deaths are reported, regardless the cause.

For each outpatient SAT, reasons for switch are reported; SAT discontinuation are highlighted in bold. Ten patients experienced SAT discontinuation, among them: 6 patients did not shown relapses (time of follow-up after discontinuation is reported in brackets; 2 patients experienced recurrence of infection and resumed SAT which is still ongoing; 2 patients experienced recurrence and death nonrelated to PVGI.

AMC, amoxicillin/clavulanate; CF, clinical failure; CIP, ciprofloxacin; CLI, clindamycin; CRO, ceftriaxone; DAL, dalbavancin; DAP, daptomycin; DOX, doxiciclin; ERT, ertapenem; FU, follow-up; i.v., intravenous; LF, laboratory failure; LVX, levofloxacin; LZD, linezolid; MIN, minocycline; NA, not available; ORT, oritavancin; ORT, oritavancin; Ph, photosensitive drug eruptions; RF, radiological failure; RIF, rifampicin; S, simplification; SAT, suppressive antimicrobial therapy; Stop, SAT discontinuation; STX, trimethoprim/sulfamethoxazole; TEI, teicoplanin.

Among 17/25 (68%) patients with an identified pathogen, the most common targeted SAT regimens were: amoxicillin/clavulanate (5), trimethoprim/sulfamethoxazole (4), teicoplanin (3), tetracycline (2), ertapenem (3), ceftriaxone (1). Among 8/25 (32%) patients without pathogen identification, SAT regimens included: amoxicillin/clavulanate (5), trimethoprim/sulfamethoxazole (3), tetracycline (3), quinolones (2), ceftriaxone (1). The decision to initiate intravenous SAT in 14 patients was made due to the absence of suitable oral alternatives, swallowing difficulties, and/or clinical or radiological worsening during oral SAT. Notably, 7 of these patients showed clinical improvement after switching to intravenous SAT following failure of oral therapy.

Combination therapy was used in 8/25 (32%) patients: 3 with GPB infections and 5 with unknown etiology. Only 1 patient received rifampin in combination therapy.

Six out of 25 (24%) patients received LAA for suspected (4) or confirmed (2) GPB. Dalbavancin was the most used LAA. LAA was generally adopted in combination regimens (5/6 patients, 83%). One patient was treated with weekly 500 mg dalbavancin, while others received 1500 mg doses with variable intervals and a mean of 4 administrations.

All recorded drug adverse events were reported in Table 2. For the analysis of drug adverse events, a total of 61 single antibiotic regimens were considered. Ten adverse events were reported in 8 patients, with 5 requiring a change in the SAT regimen. Trimethoprim/sulfamethoxazole had the highest rate of side effects (3/10, 30%). Renal toxicity was observed in 3 cases; all associated with trimethoprim-sulfamethoxazole. One case of hepatotoxicity occurred in a patient receiving ceftriaxone. Cutaneous adverse reactions included a rash linked to clindamycin and photosensitivity reactions associated with minocycline and doxycycline (2 cases in total). Nausea was noted in 1 patient treated with rifampin. Additionally, 1 patient receiving oritavancin experienced tremors, while another developed neutropenia during ertapenem therapy.

A total of 11 treatment failures, due to radiological/clinical/laboratory reason were identified among all antibiotic regimens, requiring a modification or switch in therapy (Table 2), notably 1 patient experienced more than 1 treatment failure.

The highest treatment failure rate was observed with regimen containing amoxicillin/clavulanate (8/11; 73%), followed by trimethoprim/sulfamethoxazole (2/11; 18%) and doxycycline (2/11; 18%). Seven patients experienced early failure (within 3 months from the start of SAT), due to radiological (5) or clinical-laboratory progression (2).

### Follow-up

A PET/CT scan was performed in 17/25 (68%) patients to monitor therapeutic response. A CT scan or ultrasound was used in 3/25 (12%) and 1/25 (4%) patients, respectively. The median time between the diagnostic PET/CT and the first follow-up scan (PET/CT FU) was 150 days [IQR 60–180]. Early PET/CT FU (within 3 months from diagnosis) was significantly associated with treatment failure, requiring a 2nd/3rd antibiotic regimen (83% vs 27% *P* value .04).

### Treatment Discontinuation

In 10 patients (40%), treatment is ongoing. These patients were considered improved while on SAT, having achieved infection control and resolution of clinical signs and symptoms, although complete radiological resolution has not yet been observed.

Moreover, we identified a subgroup of patients who discontinued SAT and had at least 6 months follow-up. Ten out of 25 (40%) patients discontinued SAT, due to clinical and/or radiological improvement (8) or personal decision (2), with a median treatment duration of 11 months (IQR 336 [286–447] days).

Six of these ten patients remained relapse-free after at least 6 months of follow-up (median of 12 [8–48] months) and were considered cured. Notably, 3 patients among them had previously received LAA. The remaining 4 patients experienced relapse and resumed treatment: 2 of them are currently undergoing treatment and the other 2 patients died from unrelated causes. In 1 patient the same pathogen was isolated; 1 patient had persistently negative blood culture and for the remaining 2 patients the microbiological data were unavailable. One patient relapsed due to early SAT discontinuation for personal decision. Among the 4 patients that relapsed, all presented with early and diagnosed PVGI, 3 patients had complicated PVGI due to abscess at onset and microbiological isolates were 2 GNB and 2 GPB. Among the 6 patients that discontinued SAT without relapse: 3 patients had early PVGI, 4 patients had diagnosed PVGI, 3 patients had complicated PVGI due to abscess at onset; microbiological isolates were available in 3 patients only (2 GNB and 1 GPB).

## DISCUSSION

The main results of our study are: the efficacy of SAT in achieving infection control and clinical improvement in 10 (40%) patients, in whom SAT is ongoing; the importance of monitoring SAT for drug-related adverse events (10) and treatment failures (11); the successful discontinuation of SAT in 6 (24%) patients, who did not experience relapses after long-term follow-up.

The demographic characteristics of our cohort are consistent with previously published literature, showing a prevalence of male patients, older age and multiple comorbidities [11].

Due to the lack of a universally accepted PVGI definition, the MAGIC consensus proposed diagnostic criteria based on 3 categories: clinical/surgical, radiological and laboratory [3]. Minor criteria such as fever and elevated inflammatory markers were frequently detected in our cohort (60% and 84%, respectively), while blood cultures were positive only in 67% of the cases in which they were performed. Moreover, the negativity of blood cultures allowed exclusion of PVGI diagnosis in 5 patients with dubious radiological and clinical criteria. Several limitations of the MAGIC criteria, however, have been reported: for example, postoperative bacteremia should raise suspicion for PVGI even in the absence of other diagnostic criteria [18] and clinical signs attributed to infection may stem from noninfectious causes.

Pathogen identification was successful in only 68% of patients in our cohort, a rate lower than previously reported [10]. Negative blood cultures may be due to prior antibiotic treatment or infection of the extraluminal graft surface or late-onset infections [4]. According to the literature, early infections are frequently caused by GNB or *S. aureus* [3, 6], while coagulase-negative staphylococci are commonly associated with late infections. However, we observed greater heterogeneity, with late PVGIs caused by streptococci, enterococci or polymicrobial infections, in addition to GNB and *S. aureus*. Notably, we reported 1 immunocompromised patient with *Salmonella enteritidis* and another patient who underwent surgery with a polymicrobial infection including *Actinomyces turicensis*—both uncommon causes of PVGI [19–21].

There is no “gold standard” for PVGI diagnosis [4]. CT scan remains the first-line investigation, due to easy access and wide diffusion, although interpretation can be challenging in the early postoperative period [22, 23]. Signs of suspicion for PVGI were identified by CT scan in 65% of our cohort; however, we observed 5 patients with negative CT scan but endograft hyper uptake on PET/CT scans. This represents a promising exam for both diagnosis and follow-up of PVGI, though not universally available [24–26]. Moreover, no SUVmax value threshold is validated to reliably distinguish between inflammation and infection [23] and the optimal timing for PET/CT FU is not established [27]. In our study, 17 patients performed PET/CT FU scans, which supported SAT regimen changes in 4 patients and SAT discontinuation in 4 patients. Early follow-up imaging was often associated with apparent radiological worsening or no improvement, prompting changes in SAT. We hypothesize that longer observation periods may be necessary to detect radiological improvement; therefore, conducting PET/CT FU scans earlier than 6 months after baseline may lead to misleading or inconclusive results.

Clinical studies on PVGI report great heterogeneity in SAT regimens [10, 28]. Antibiotics with biofilm-activity—such as rifampin, quinolones, linezolid or daptomycin—are recommended [10, 29]. In our cohort, intravenous daptomycin was used only for a limited period and linezolid was rarely used due to limited tolerability [30]. We adopted rifampin combination therapy in only 1 patient, primarily due to resistant strain findings or drug–drug interactions. Tetracycline demonstrated good efficacy and long-term tolerability in our cohort, consistent with literature findings [28]. Moreover, amoxicillin/clavulanate was one of the most prescribed antibiotics in our cohort (10/25 patients), adopted for PVGIs caused by GPB (3), GNB (2) or unknown pathogens (5). Fewer therapeutic options are available for GNB. Ciprofloxacin was generally effective and well-tolerated; however, quinolones should be used cautiously due to the known risk of aortic aneurysm or dissection [31]. Cotrimoxazole, with activity against both GPB and GNB, was used in 7 patients, but was associated with side effects, particularly renal impairment in older patients or at higher doses required for *S. aureus* infections.

Combination therapy should be considered in polymicrobial PVGI, unknown pathogen, or difficult-to-treat strains. However, this approach is often limited by poor patient compliance and an increased risk of adverse effects. Therefore, only 8 patients (32%) in our cohort received combination therapy, most involving a LAA combined with oral SAT.

Reports of off-label use of LAAs in cardiovascular infections are increasing, thanks to good tissue penetration, efficacy against biofilms and a strong activity against resistant *Staphylococcus* and *Enterococcus* strains [32–34]. Six patients in our cohort received LAAs as sequential therapy. Unfortunately, therapeutic drug monitoring (TDM), which is recommended in long-term treatment [35], was not routinely available in our hospital.

Finally, discontinuing SAT in PVGI remains challenging and requires careful patient selection. While 6 patients were considered cured, 4 experienced relapse and required resumption of SAT. Therefore, long-term follow-up is essential.

The present study has several limitations that affect the generalizability of the results. These include its retrospective and single-center design, the limited sample size, the heterogeneity of surgical interventions, the lack of data on the causes of death in deceased patients, the possibility of bias in the assessment of treatment failure with potential overestimation of failure rates. Also, LAAs were administered without a standardized scheme in terms of dose and timing, and TDM was not performed in our cohort. Moreover, relapse and reinfection could not be reliably differentiated in the absence of microbiological data. However, we included only patients with a homogeneous definition of PVGI, all of whom were managed by a dedicated multidisciplinary team. In our cohort, 6 patients successfully discontinued SAT without relapse; although the number of patients is limited, the long follow-up period after discontinuation is a strength of our study.

## CONCLUSIONS

Our findings support SAT as a viable option for patients with PVGI who are at high surgical risk. LAA is a possible alternative to oral SAT, although further supporting data are needed. Moreover, PET/CT scan plays a key role in both diagnosis and follow-up; however, the optimal timing for evaluation remains unclear.

Larger multicenter studies are needed to assess the long-term efficacy and optimal duration of SAT. Finally, a multidisciplinary approach should always be adopted—encompassing specialists in infectious diseases, surgery, microbiology, radiology, nuclear medicine, and, where possible, clinical pharmacology— to establish expert consensus and to optimize the use of new therapeutic strategies.

## Supplementary Material

ofag327_Supplementary_Data

## Supplementary Data

Supplementary materials are available at *Open Forum Infectious Diseases* online. Consisting of data provided by the authors to benefit the reader, the posted materials are not copyedited and are the sole responsibility of the authors, so questions or comments should be addressed to the corresponding author.
