# Supplementary material for: Long-Term Suppressive Antimicrobial Therapy in Prosthetic Vascular Graft Infection: A Retrospective Evaluation of a Cohort of Patients Enrolled at Tor Vergata Hospital in Rome
Source: Open Forum Infect Dis. 2026 Jun 5;13(6):ofag327. doi: 10.1093/ofid/ofag327 (PMC13263525; doi:10.1093/ofid/ofag327)
Supplement: ofag327_Supplementary_Data [file ofag327_supplementary_data.zip › Supplementary material.docx]

**Supplementary material**

**Suppl. Tab. 1.** Characteristic of the cohort that underwent SAT, overall and after stratification for continuity of treatment (lifelong SAT vs SAT discontinuation).

|  | Overall  (25) | Lifelong SAT (15) | SAT discontinuation  (10) |
| --- | --- | --- | --- |
| Male n (%) | 21 (84) | 13 (87) | 8 (80) |
| Age (years) | 72 [62-78] | 76 [63-78] | 66 [59-73] |
| Age > 65 years n (%) | 16 (64) | 10 (67) | 6 (60) |
| CCI | 3 [3-6] | 4 [3-7] | 3 [3-5] |
| Type II diabetes mellitus n (%) | 6 (24) | 3 (20) | 3 (30) |
| Pulmonary n (%) | 5 (20) | 2 (13) | 3 (30) |
| Renal n (%) | 6 (24) | 5 (33) | 1 (10) |
| Cardiovascular n (%) | 19 (76) | 12 (80) | 7 (70) |
| Hepatological n (%) | 2 (8) | 1 (7) | 1 (10) |
| Neurological n (%) | 1 (4) | 0 | 1 (10) |
| Thoracic/Abdominal/Thoraco-abdominal graft n (%) | 7 (28) / 15 (60) / 3 (12) | 5 (33) / 8 (53) / 13 (2) | 2 (20) / 7 (70) / 1 (10) |
| Emergency n (%) | 12 (48) | 7 (47) | 5 (50) |
| Early n (%) | 14 (56) | 7 (47) | 7 (70) |
| Suspected (%) | 11 (44) | 9 (60) | 2 (20) |
| Abscess n (%) | 12 (48) | 6 (40) | 6 (60) |
| Increase in inflammatory indices n (%) | 21 (84) | 13 (87) | 8 (80) |
| Fever n (%) | 15 (60) | 9 (60) | 6 (60) |
| Pain n (%) | 12 (48) | 8 (53) | 4 (40) |
| Pathogen unknown n (%) | 8 (32) | 5 (33) | 3 (30) |
| Enterococci spp n (%) | 1 (4) | 0 | 1 (10) |
| Streptococci spp n (%) | 3 (12) | 3 (20) | 0 |
| *Staphylococcus aureus* n (%) | 4 (16) | 2 (13) | 2 (20) |
| Coagulase-negative Staphylococci n (%) | 2 (8) | 2 (13) | 0 |
| Gram negative bacteria n (%) | 7 (28) | 3 (20) | 4 (40) |
| Polymicrobial n (%) | 2 (8) | 2 (13) | 0 |
| Long-acting antibiotic n (%) | 6 (24) | 2 (13) | 4 (40) |

Quantitative variables are presented as median (interquartile range); categorical variables are presented as percentages. CCI: Charlson Comorbidity Index; SAT: suppressive antimicrobial therapy. PVGI were classified as early/late according to *Lyons OT et al. Diagnosis of Aortic Graft Infection: A Case Definition by the Management of Aortic Graft Infection Collaboration (MAGIC). Eur J Vasc Endovasc Surg. 2016.*

**Suppl. Fig. 1** Follow-up and outpatient assessment.


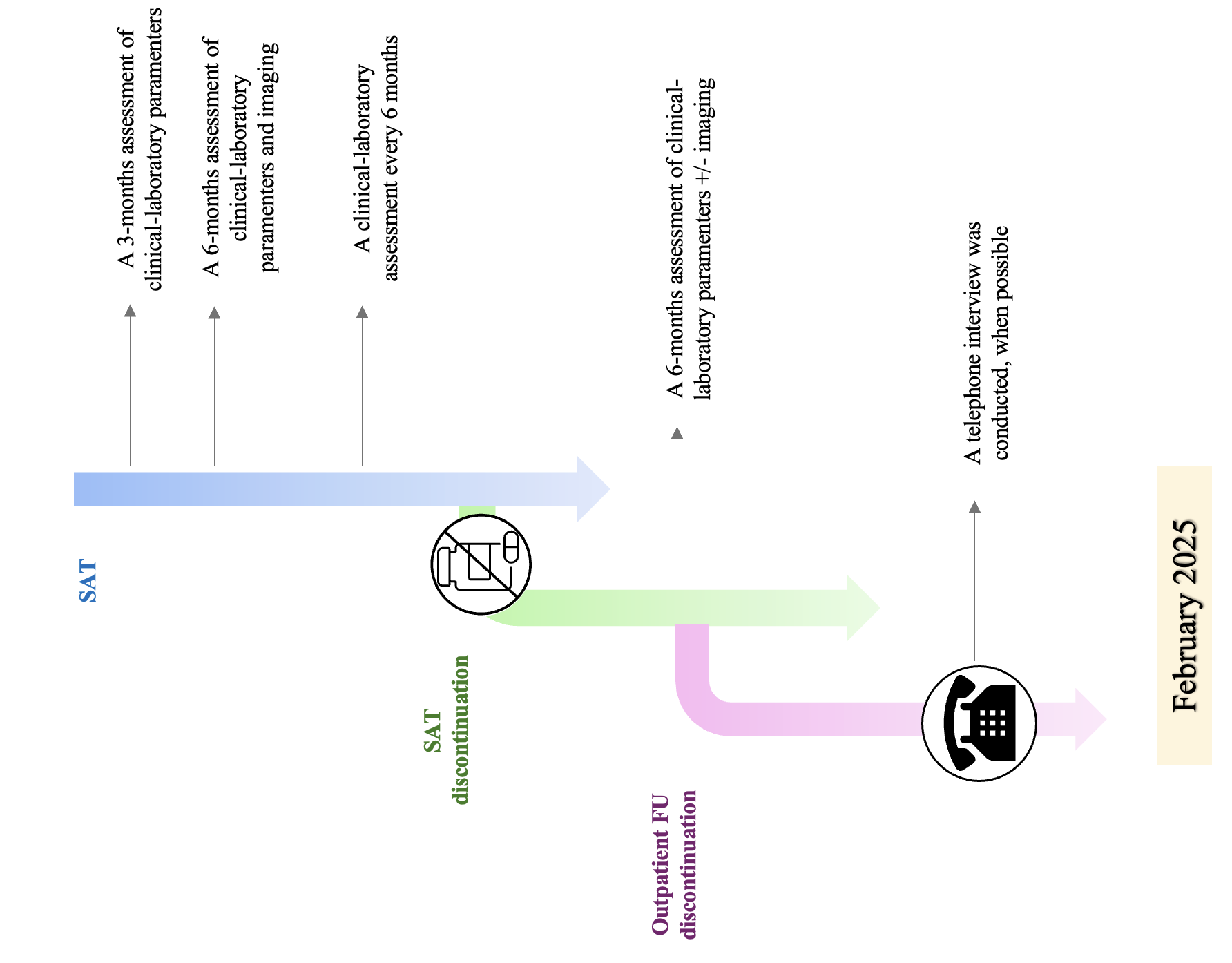


FU: follow-up; SAT: suppressive antimicrobial therapy.

Outcomes were retrospectively assessed based on the most recent available outpatient evaluation at the date of February 2025. For patients who had discontinued outpatient follow-up, telephone interviews were conducted, when possible, to obtain up-to-date clinical information.

**Alt text**: Graphical flowchart showing SAT monitoring steps and outpatient assessment timeline.

**Suppl. Fig. 2.** Flowchart of the selecting process of patients with prosthetic vascular graft infection (PVGI).


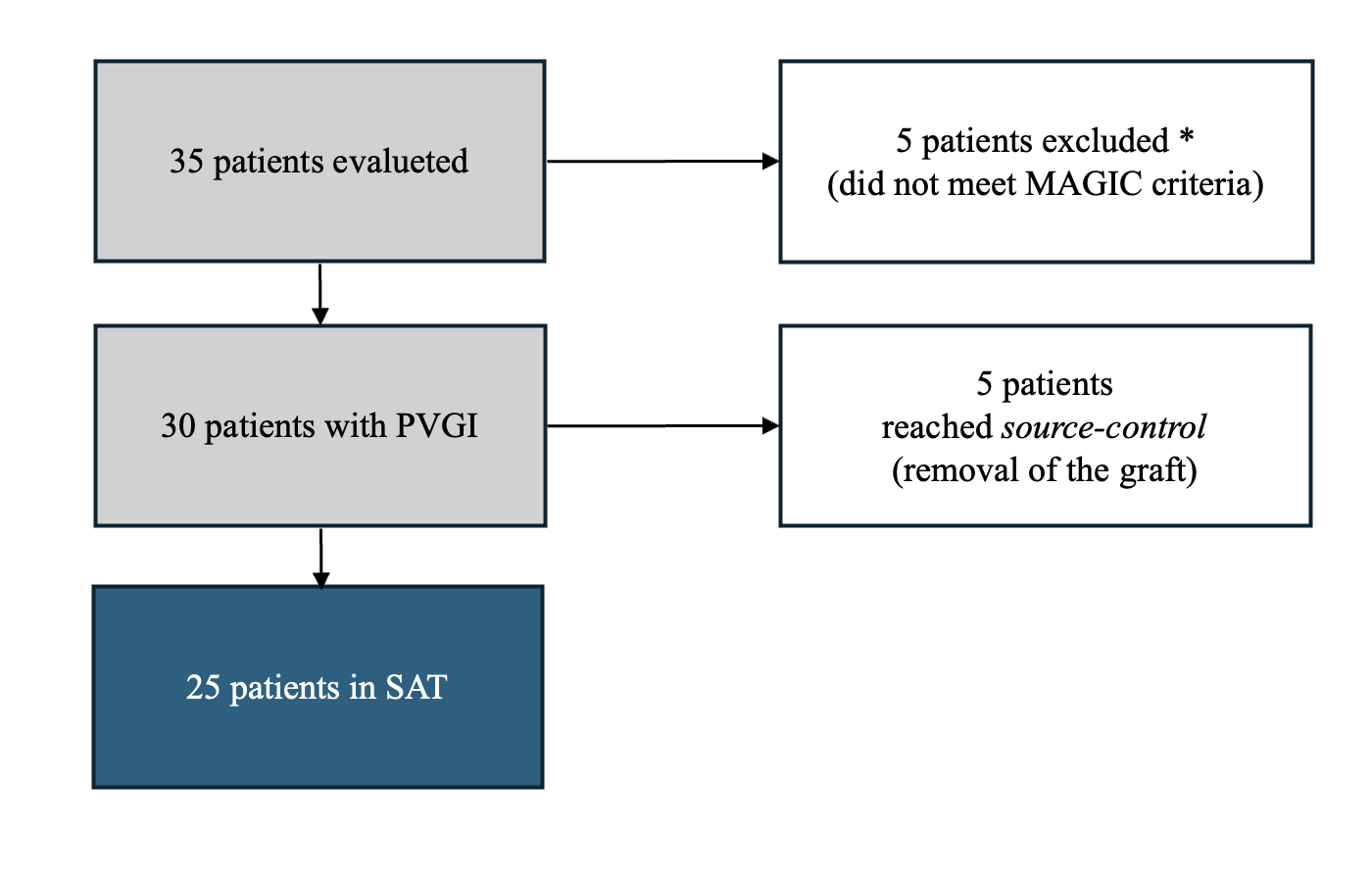


PVGI: Prosthetic vascular graft infection; SAT: suppressive antimicrobial therapy.

PVGI were included according to MAGIC definitions *(Lyons OT et al. Diagnosis of Aortic Graft Infection: A Case Definition by the Management of Aortic Graft Infection Collaboration (MAGIC). Eur J Vasc Endovasc Surg. 2016).*

*P.V. and M.G.G. presented with increased metabolic activity of the graft at the PET/CT scan but in absence of clinical-laboratory sign of infection as if from inflammation rather than infection. D.C.G presented with fever and increase in inflammation indices, but the absence of metabolic activity excluded the infection of the graft. S.F. and C.A. presented with fever, increase in inflammation indices and appearance of peri-graft fluid at CT scan in the early postoperative period (within 30 days of graft implantation), which were not confirmed at further analysis. All patients performed serial blood cultures, which resulted negative, and no pathogens were identified.

**Alt text:** Graphical flowchart of the selecting process of the population.
