# Supplementary figures and images for: Long-Term Suppressive Antimicrobial Therapy in Prosthetic Vascular Graft Infection: A Retrospective Evaluation of a Cohort of Patients Enrolled at Tor Vergata Hospital in Rome
Source: Open Forum Infect Dis. 2026 Jun 5;13(6):ofag327. doi: 10.1093/ofid/ofag327 (PMC13263525; doi:10.1093/ofid/ofag327)

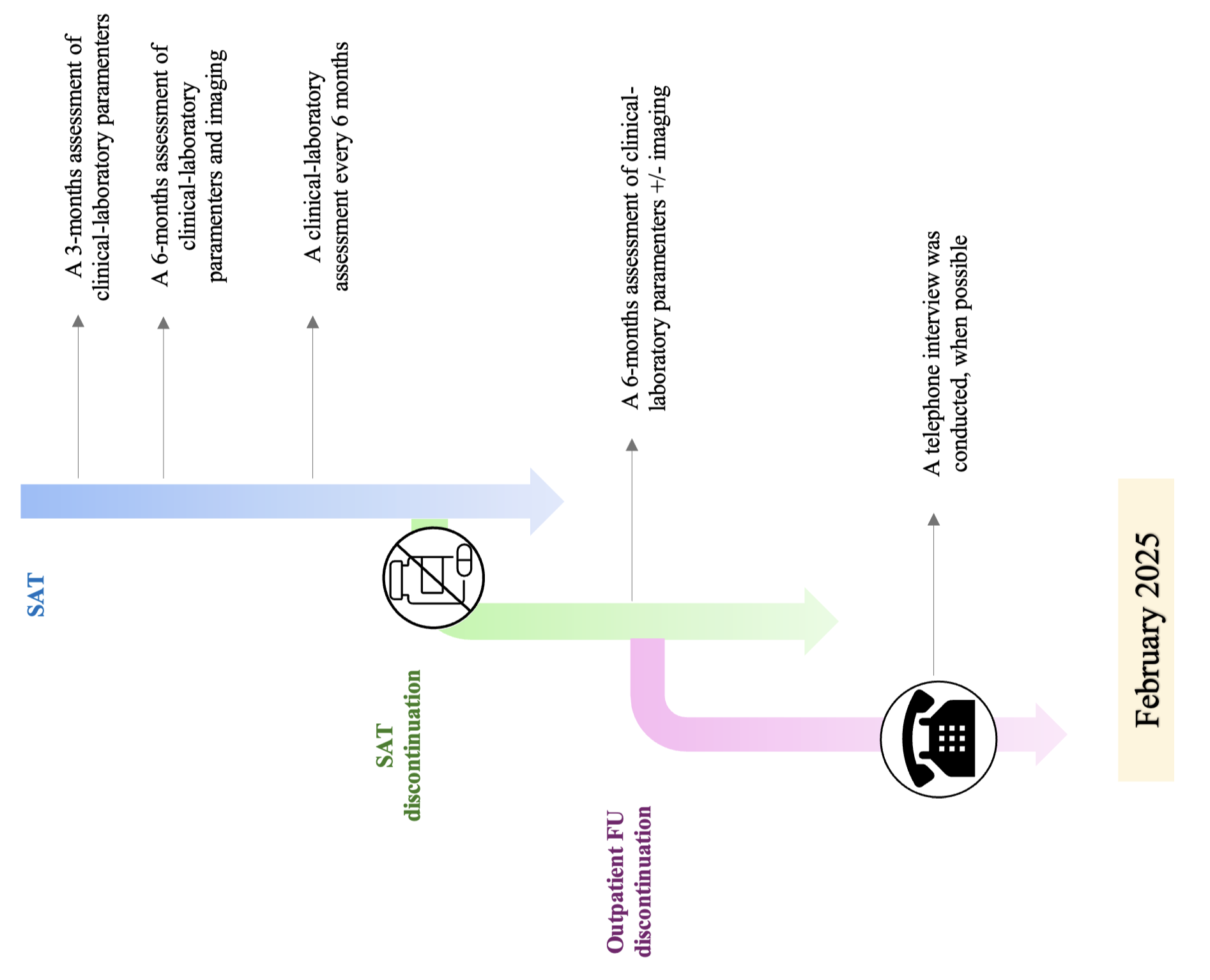

Supplement: ofag327_Supplementary_Data [file ofag327_supplementary_data.zip › s1.tiff]

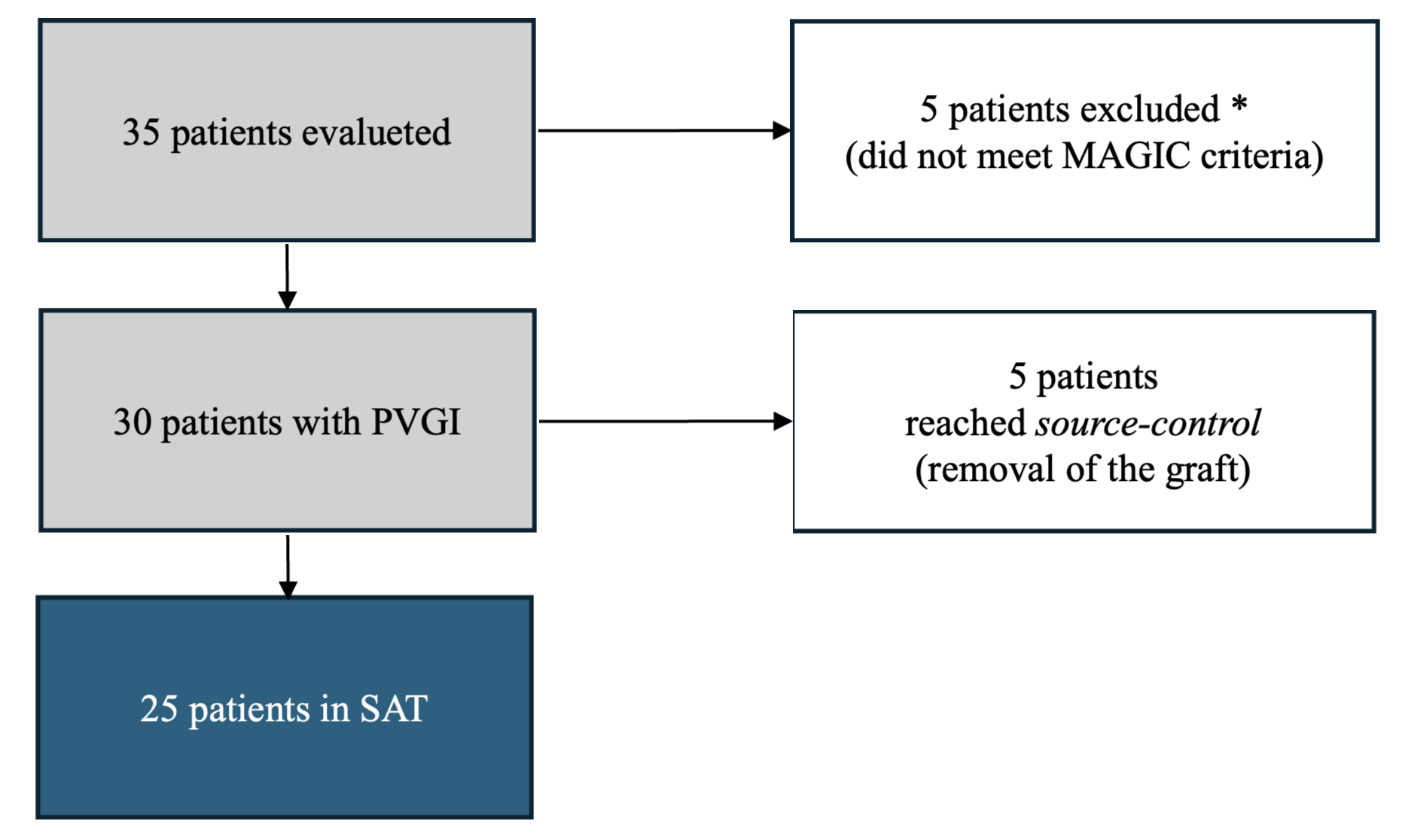

Supplement: ofag327_Supplementary_Data [file ofag327_supplementary_data.zip › s2.tiff]
